# Mechanical properties of thermoformed and direct-printed aligner materials after immersion in 37 °C water: a 14-day *in vitro* study

**DOI:** 10.1038/s41598-026-36723-8

**Published:** 2026-01-21

**Authors:** Rodrigo Oyonarte, Isabel Margarita Lagos, Francisca Vidaurre L., Tomás Parada B., Alberto del Real, Soonho Jang, Harim Jeong, Jiho Lee, Jinhong Min, Tarek M. Elshazly, Jung-Yul Cha, Hoon Kim

**Affiliations:** 1 Facultad de Odotología, Universidad de los Andes, Monseñor Alvaro del Portillo, Las Condes, Santiago, 12455 Chile; 2 Graphy Inc., Graphy R&D Center, Seoul, 08501 Republic of Korea; 3 Oral Technology Department, Dental School, University Hospital Bonn, Welschonnenstr. 17, 53111 Bonn, Germany; 4 Department of Orthodontics, Institute of Craniofacial Deformity, College of Dentistry, Yonsei University, 50 1 Yonsei ro, Seodaemun gu, Seoul, 03722 Korea; 5 Research Institute of Agriculture and Life Sciences, College of Agriculture & Life Sciences, Seoul National University, Seoul, 08826 Republic of Korea

**Keywords:** Clear aligners, 3D printing, Thermoforming, Mechanical properties, Water aging, Tensile strength, Elongation at break, Health care, Materials science, Medical research

## Abstract

This study compared the mechanical properties of direct-printed dental aligner materials made from 3D-printed resins TC-85, TR-07, and TA-28 with those of two conventional thermoformed materials—Zendura-A and Zendura-Flx—to evaluate their performance under simulated physiological conditions. Test specimens were immersed in a 37 °C water bath for 12 different durations: 0, 5, and 30 min; 1, 3, 6, and 9 h; and 1, 3, 7, and 14 d. Tensile tests were performed using a universal testing machine (Zwick Z010, Zwick, Ulm, Germany) to measure the Young’s modulus (MPa), elongation at break (%), and tensile force (N) at strains of 1%, 2%, and 3%. After 14 d of immersion, TC-85, TA-28, and TR-07 exhibited forces in the range of 4.04–7.24 N at 1% strain and 7.30–13.48 N at 3% strain, while Zendura A and Zendura FLX exhibited forces of 26.26–32.91 N at 1% strain and 32.91–65.23 N at 3% strain. The Young’s modulus and UTS results exhibit a trend similar to that of the tensile force. Direct-printed aligners exhibited a 25.3% (TC-85) increase in elongation at break after 30 min, whereas thermoformed aligners exhibited a 5.5% reduction. Direct-printed resins, such as TC-85, TA-28, and TR-07, with temperature-responsive viscoelastic behavior, exhibited statistically significant differences from thermoformed aligner materials, delivering lower mechanical loads that could favor a more suitable orthodontic force profile for clear aligners.

## Introduction

The demand for clear aligner therapy (CAT) in orthodontic treatment has recently increased, particularly among adults with high aesthetic demands^1^. Consequently, orthodontists are increasingly producing aligners in-house, primarily using thermoformed materials to optimize efficiency and reduce manufacturing time. Thus, efforts to develop advanced materials using computer-aided design/computer-aided manufacturing (CAD/CAM) technologies have led to the development of direct 3D-printed aligners^2,3^.

The most commonly used materials for thermoformed aligners are glycol-modified polyethylene terephthalate (PETG), thermoplastic polyurethane (TPU), and multilayer hybrid composites^2^. PETG is an amorphous copolymer with high transparency, aesthetics, formability, and dimensional stability. However, its viscoelastic nature leads to rapid strength loss, which can compromise the effectiveness of the aligner treatment^4–6^. TPU, which is known for its elasticity and abrasion resistance, maintains light and constant forces but can become opaque after thermoforming and deteriorate with prolonged use^2,4,5,7,8^. Water absorption leads to degradation of mechanical properties with intraoral use^9,10^.

The thermoforming of the printed models requires time and effort^2^. One study found that thermoformed aligners undergo dimensional variations, including a 35% reduction in thickness and changes in transparency, water absorption, hardness, and elastic modulus^11,12^. Direct 3D printing with biocompatible materials has gained popularity for increasing the precision and efficiency. The direct-printing method offers higher dimensional accuracy and better control over the aligner design and thickness, resulting in aligners with higher fit accuracy^1,13,14^. It may also reduce the manufacturing time with its model-less process^15–17^.

To achieve effective tooth movement and successful orthodontic treatment with CAT, aligners must apply a light and constant force while remaining durable and flexible. This ensures that the orthodontic force applied to the teeth remains consistent and effective over time. Aligners must resist intraoral wear but not be rigid enough to interfere with force application, ensuring that they remain within the biomechanically comfortable elastic range^18^. While extensive literature exists on the mechanical properties of thermoformed materials^5,11,12,19^, there is relatively limited research on direct-printed aligners. Yet, the direct-printed aligner resin TC-85 (Tera Harz Clear), which is also included in this study, has been the most widely studied with investigations into its viscoelasticity, dynamic mechanical properties, temperature-responsive physical and biomechanical performance, biocompatibility and cytotoxicity, as well as its shape memory effects^1–3,9,13,15–17,20–28^.

Although several studies have investigated the mechanical behavior of thermoformed materials and direct-printed resins used in orthodontic aligners, there is a lack of research on how these materials perform when exposed to liquid immersion, which simulates the oral environment. A recent study evaluated the mechanical changes in clear aligners caused by exposure to different liquids^10^. However, they only considered a 24-h immersion period. No studies have specifically investigated the effects of prolonged immersion in water at 37 °C, which simulates the typical temperature of the human mouth, on the mechanical properties of these materials. This represents a critical gap in the literature, because aligners are exposed to oral fluids at body temperature over extended periods. Therefore, it is essential to comprehensively analyze the mechanical properties of the aligner materials, focusing on their potential deterioration after 14 d of immersion at 37 °C, and to simulate long-term exposure of aligners to intraoral conditions, with a focus on temperature and moisture.

This study aimed to evaluate the mechanical properties of the most widely used direct-printed shape memory aligner resin, TC-85 alongside two newly introduced biocompatible resins, TR-07 and TA-28, and to evaluate their behaviors relative to conventional thermoformed materials, including single-layer TPU and multilayer TPU. All materials were assessed under controlled thermo-hydric conditions, using 37 °C water immersion across multiple time points over a 14-day period to capture their time-dependent mechanical responses.

## Materials and methods

### Specimen preparation

Clear aligner resins, TC-85, TR-07, and TA-28 (Graphy Inc., Seoul, South Korea), were printed into dumbbell-shaped specimens with a modified thickness of 0.5 mm (ASTM D638-Type V) using a DLP 3D printer (Uniz NBEE, Uniz, San Diego, CA, USA) with a layer thickness of 100 μm. They were post-cured using a curing machine (Tera Harz Cure, Graphy, Seoul, South Korea) equipped with oxygen inhibition. The direct-printed samples were cured under nitrogen condition at UV level 2 for 20, 25, and 15 min for TC-85, TR-07, and TA-28, respectively.

The nominal thickness of ASTM D638 Type 5 tensile specimen was modified from the standard 3.2 mm to 0.5 mm to reflect clinically relevant aligner thickness after fabrication and to enable force measurements within a clinically effective orthodontic range. In addition, commercially available thermoforming aligner sheets typically exhibit nominal thicknesses ranging from 0.5 mm to 0.89 mm prior to thermoforming, making fabrication of standard 3.2 mm specimens impractical.

**Fig. 1:**
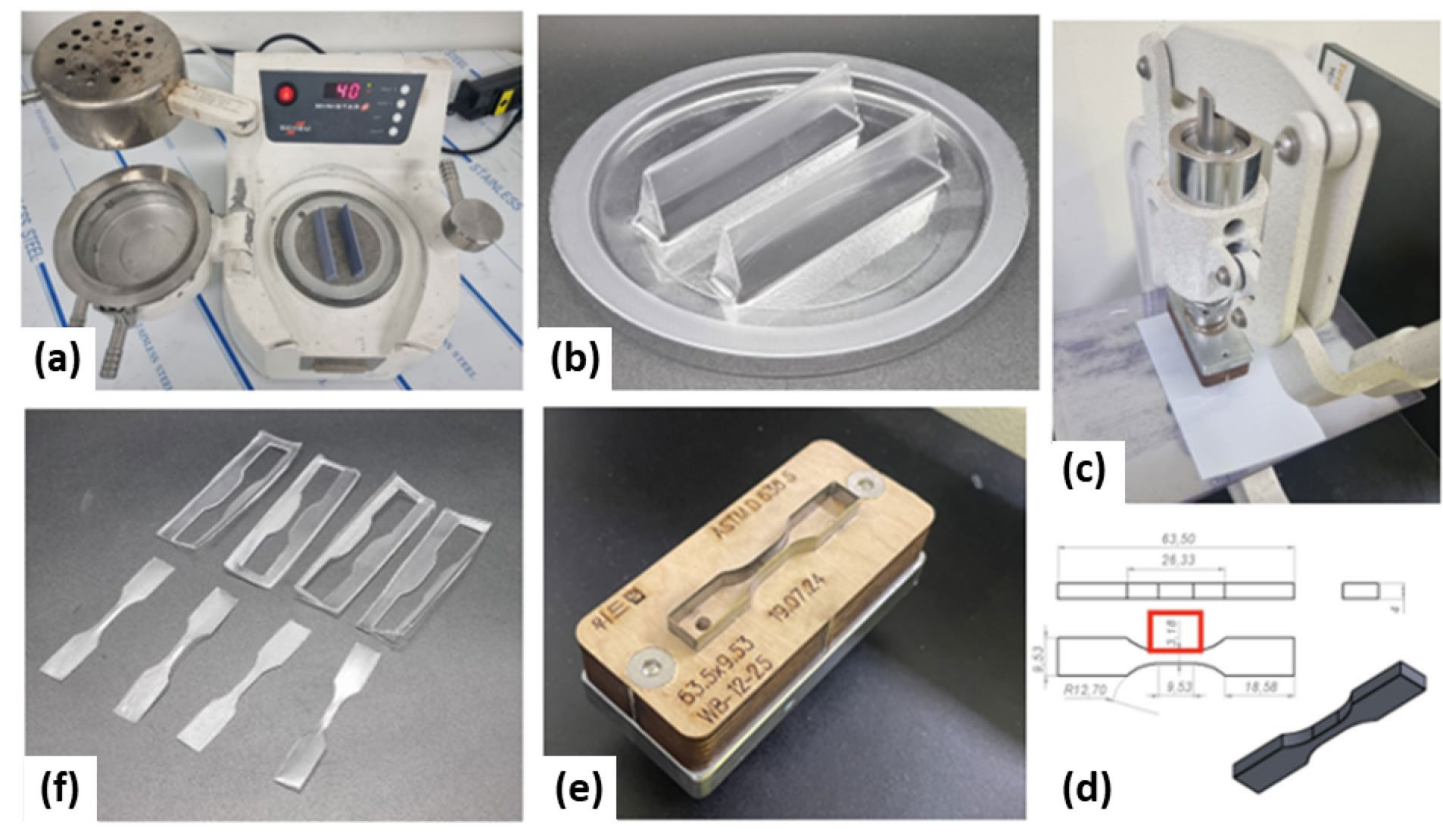
**(a)** thermoforming machine (MINISTAR S, Scheu Dental, Germany), **(b)** thermoformed sheet on simulation model, **(c)** punching unit for ASTM D638-5 Specimen (WL1200J, WITHLAB Co., Ltd, South Korea), **(d)** specimen dimensions for ASTM D638 Type 5 test standard, **(e)** custom-designed cutting press for ASTM D638 Type 5, and **(f)** trimmed thermoformed specimens ready for tensile testing.

For thermoformed aligners, Zendura A (single-layer TPU) and Zendura FLX (multi-layer TPU) circular sheets (0.76 mm thickness x 125 mm; Bay Materials, USA) were thermoformed using a vacuum-forming unit (Ministar S, Scheu-Dental GmbH, Germany). To reflect clinically relevant manufacturing conditions, a standardized maxillary central incisor model was designed following a previously established protocol^2^. The model incorporated an incisal edge thickness of 2 mm, a height-of-contour thickness of 8.5 mm, a clinical crown height of 7 mm, and an overall tooth height of 20 mm. The standardized model was fabricated using a LCD 3D printer (Uniz 4 K, Uniz, USA) with a dental model resin (S-100, Graphy Inc., South Korea). The thermoformed sheets were subsequently cut into ASTM D638 Type V tensile specimens using a punching machine (WithLab Co.,Ltd, South Korea) equipped with a custom-designed cutting press, in accordance with ASTM D638 Type 5 testing standards. The preparation workflow is shown in Fig. 1.

### Water-bath immersion

The test specimens were immersed in a distilled water bath at 37 °C for durations of 0 min, 5 min, 30 min, 1 h, 3 h, 6 h, 9 h, 1 d, 3 d, 7 d, and 14 d. Because each clear aligner is typically prescribed for 1–2 weeks of wear to achieve its intended tooth movement before replacement, the evaluation period was set up to 14 days.

Immersion was performed in a digitally controlled laboratory oven (Lab Companion, South Korea) equipped with active temperature regulation, maintaining the water bath at 37 °C throughout the entire immersion periods. The immersed samples were measured for their dimensions and tested immediately upon removal without drying.

A total of 385 specimens (5 materials x 11 immersion conditions x 7 replicates) were prepared and tested in this study. The sample size (*n* = 7 per group) was selected based on established experimental practices in preclinical polymer science and material behavior studies, where replicated 5–10 specimens are commonly used to for evaluate mechanical behavior and comparative material responses.

### Tensile testing

Prior to mechanical testing, the final post-manufacturing thickness and width of each specimen were measured using a digital vernier caliper. The measured cross-sectional area of each specimen was used for stress calculations to ensure valid interpretation of material behavior. Mean final post-manufacturing thickness values for all materials are summarized in Table 1.

The time-dependent changes in the mechanical properties of the different transparent orthodontic materials under simulated thermo-hydric aging conditions were assessed using a universal testing machine (Zwick Z010; Zwick, Ulm, Germany) following the ASTM D638-Type-V test standard with crosshead speed of 5 mm/min, a gauge length of 7.63 mm with extensometers, grip separation of 32 mm, and a controlled room temperature of 25 ± 1 °C. The following properties were measured: tensile force (N) at strain levels of 1%, 2%, and 3%; ultimate tensile strength (UTS) (MPa); Young’s modulus (MPa); and elongation at break (%).

#### Justification for 1%–3% strain

The forces measured at different strains (1%, 2%, and 3%) are critical for understanding the magnitude of orthodontic force exerted by aligner during clinical use.

**Fig. 2:**
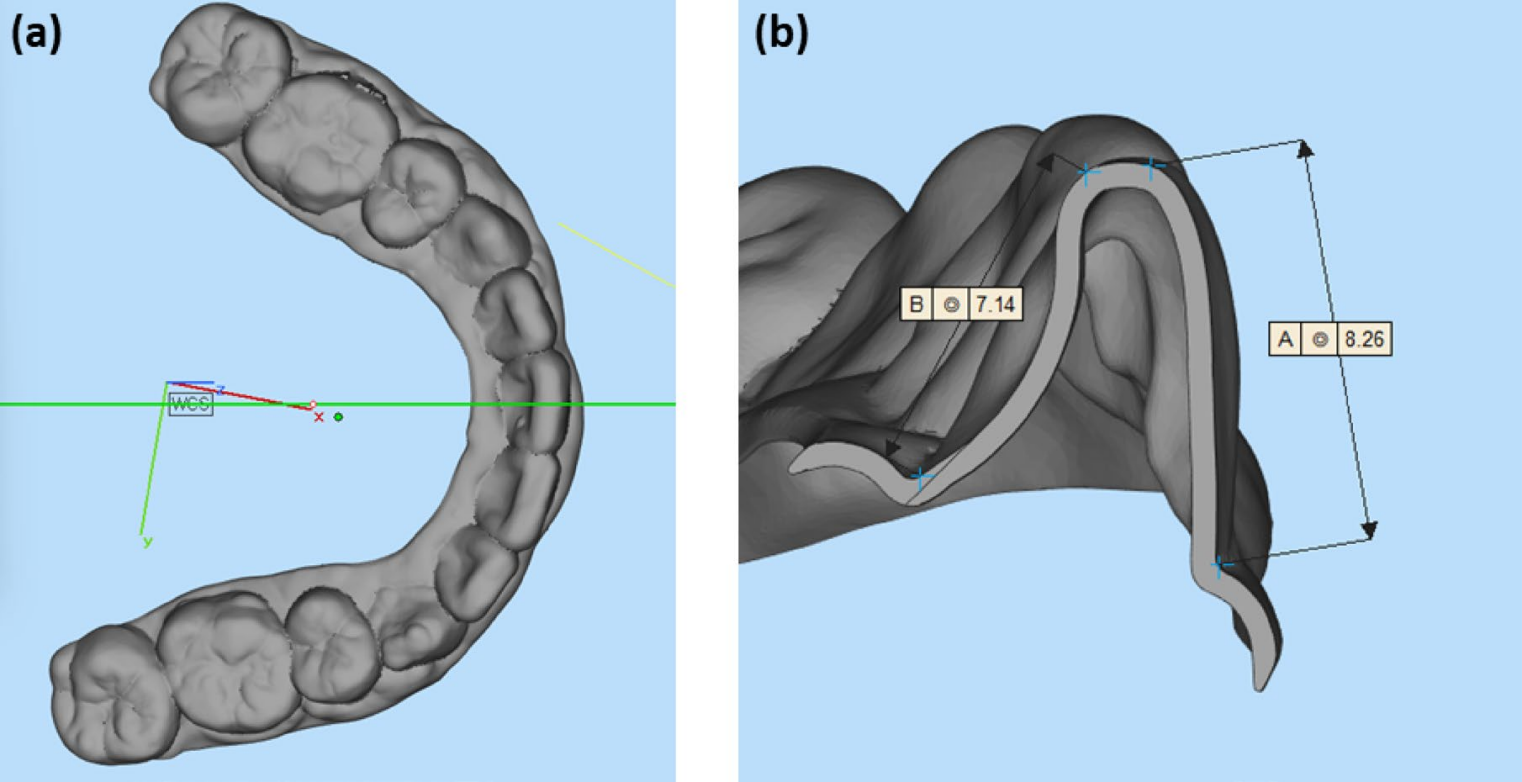
Orthodontic clear aligner dimensions measured with 3D design software, Materialise Magics 25.01. **(a)** Sample aligner with a cross-sectional line, **(b)** cross section at mandibular central incisor. (A : crown-covering aligner length at labial, 8.26 mm/B : crown-covering aligner length at lingual, 7.14 mm).

Strain levels of 1–3% were selected to reflect the clinically relevant deformation range experienced by aligners. According to previous studies^29,30^, the average length of the anterior teeth is approximately 10 mm, resulting in a corresponding aligner cross-sectional length in the anterior region of approximately 15–25 mm. Given the typical interproximal tooth movement per aligner ranging from 0.1 to 0.5 mm, the resulting maximum strain exerted on the aligner material would be approximately 2%. This estimation is consistent with values obtained in other *in vitro* studies^2,4,15^. Figure 2 illustrates representative aligner dimensions measured used to support this strain range.

Accordingly, the force levels measured at 1–3% strain were considered suitable for assessment of orthodontically relevant force magnitude range, aligning with biomechanical recommendations for orthodontic forces in the range of 0.2–2.5 N and deliver light and continuous forces within the physiological range^2,31–33^.

### Statistical analysis

Stata v18 and Prism GraphPad software were used for statistical analyses. The Shapiro–Wilk test was used to assess the normality of the sample distribution. Because the data did not follow a normal distribution, the nonparametric Kruskal–Wallis test with post hoc Dunn’s post hoc test with multiple-comparison test was used to compare the mechanical properties of the different materials across the 11 immersion periods (0 min, 5 min, 30 min, 1 h, 3 h, 6 h, 9 h, 1 d, 3 d, 7 d, and 14 d). The Friedman test was used to analyze each material under prolonged immersion in 37 °C distilled water, followed by pairwise comparisons between time points using Dunn’s test.

## Results

### Post-manufacturing thickness of aligner materials

Prior to mechanical testing, final post-manufacturing thicknesses were measured for all immersion conditions. Zendura A and Zendura FLX showed a mean final thickness of approximately 0.41 mm and 0.38 mm, respectively, whereas the 3D-printed materials TC-85, TR-07, and TA-28 exhibited mean thicknesses in the range of 0.56–0.59 mm. Detailed values are summarized in Table 1.

**Table 1:** Mean post-manufacturing thickness (mm) of clear aligner materials measured across all water-immersion conditions (0 min to 2 weeks at 37 ° C). Final thickness values were recorded immediately prior to tensile testing and used in all stress calculations.

Water Exposure Time	*n*	Material Type				
		3D Printing (Initial Design Thickness: 0.4 mm)			Thermoforming (Initial Sheet Thickness: 0.762 mm)	
		TC-85	TR-07	TA-28	Zendura A	Zendura FLX
***0 min***	7	0.5900	0.5817	0.5767	0.4040	0.4120
***5 min***	7	0.6150	0.5833	0.5583	0.4420	0.4080
***30 min***	7	0.5917	0.5850	0.5717	0.4180	0.3780
***1 h***	7	0.5833	0.5733	0.5717	0.4283	0.3460
***3 h***	7	0.5850	0.5550	0.5717	0.3780	0.3840
***6 h***	7	0.5817	0.5650	0.5450	0.4083	0.3740
***9 h***	7	0.5683	0.5500	0.5567	0.4317	0.4180
***1 day***	7	0.5767	0.5700	0.5633	0.4050	0.3560
***3 days***	7	0.5683	0.5567	0.5667	0.3950	0.3580
***7 days***	7	0.5933	0.5700	0.5683	0.3850	0.3280
***10 days***	7	0.5867	0.5567	0.5533	0.4383	0.3960
***14 days***	7	0.5783	0.5200	0.5183	0.4360	0.4120
***Total Average***	***77***	***0.5849***	***0.5639***	***0.5601***	***0.4141***	***0.3808***

### Tensile force at 1%, 2%, and 3% strain

**Fig. 3:**
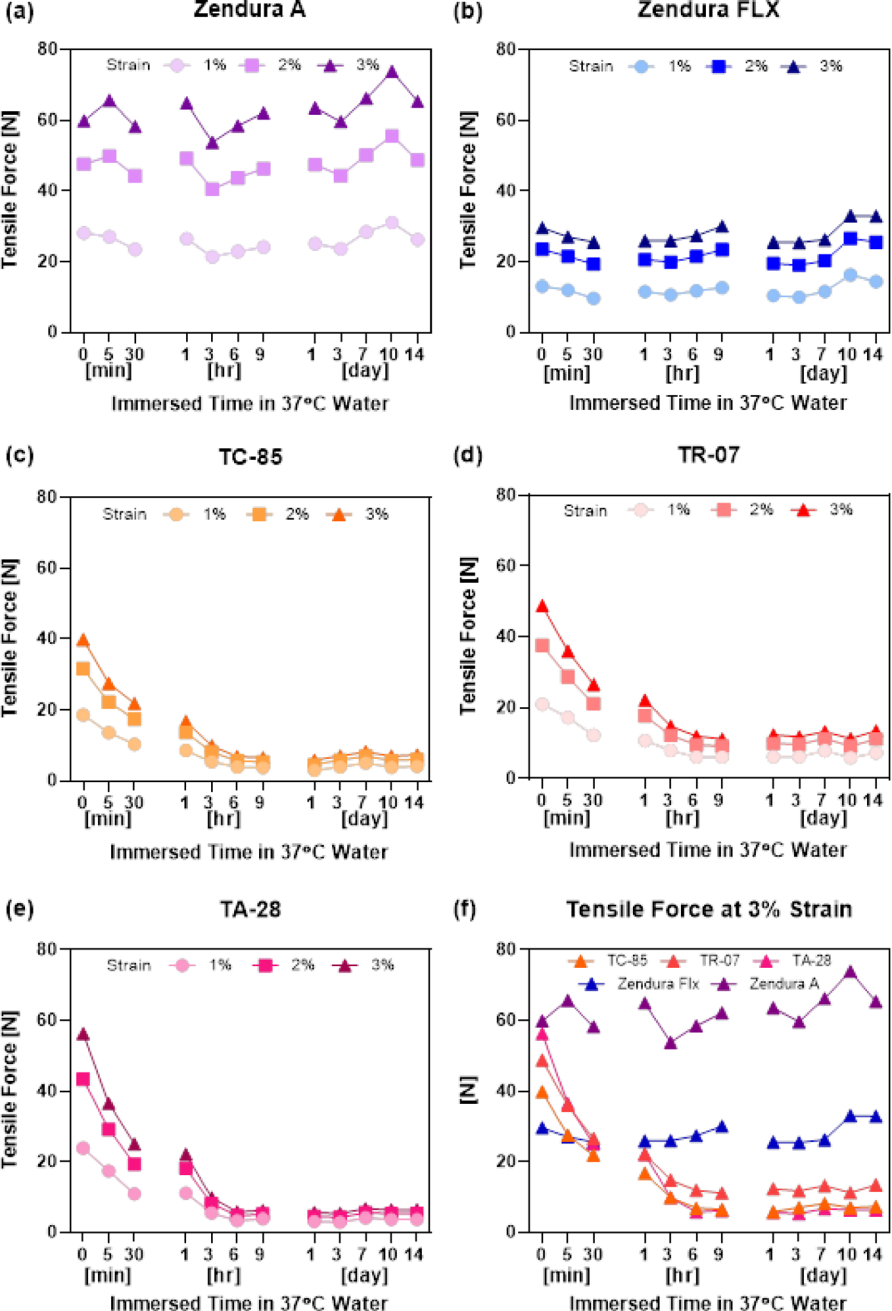
Tensile forces (N) of five different clear aligner materials at 1%, 2%, and 3% strain with respect to the immersed time in 37 °C distilled water. TFA: **(a)** Zendura A, **(b)** Zendura FLX, DPA: **(c)** TC-85, **(d)** TR-07, **(e)** TA-28, and **(f)** all five aligners at 3% strain. (TFA: thermoformed aligner, DPA: direct printed aligner).

The tensile forces (N) of direct-printed aligners (TC-85, TR-07, TA-28) and thermoformed aligners (Zendura A, Zendura FLX) were measured at 1%, 2%, and 3% strain across different water-immersion durations at 37 °C. Figure 3 illustrates the variation in tensile forces over time for all materials at 1%, 2%, and 3% strain, and Table 2 presents a detailed overview of the exact tensile forces (N) measured for each material at all immersion time points. TC-85 and TA-28 demonstrated significant reductions over time, with their lowest tensile forces recorded at later immersion times, reaching as low as 2.88 N (TC-85, 1% strain, 14 d) and 2.91 N (TA-28, 1% strain, 3 d).

**Table 2:** Tensile forces (N) at 1%, 2%, and 3% strain levels for clear aligner materials at varied water immersion times.

Strain (%)	Clear Aligner Materials	Immersed Time in 37° C Water Bath											
		0 min	5 min	30 min	1 h	3 h	6 h	9 h	1 day	3 days	7 days	10 days	14 days
***1***	***TC-85***	18.62	13.58	10.30	8.54	5.34	3.85	3.65	2.88	3.87	4.97	3.78	4.04
	***TR-07***	20.96	17.27	12.23	10.68	7.91	6.06	6.09	6.10	6.10	7.85	5.89	7.24
	***TA-28***	23.84	17.37	10.91	11.16	5.50	3.38	3.88	3.11	2.91	4.14	3.70	3.67
	***Zendura A***	28.09	27.01	23.46	26.46	21.37	22.89	24.12	25.09	23.62	28.41	31.10	26.26
	***Zendura FLX***	13.08	11.97	9.62	11.53	10.62	11.79	12.70	10.42	9.95	11.59	16.28	14.36
***2***	***TC-85***	31.58	22.16	17.39	13.66	8.10	5.63	5.34	4.54	5.83	6.91	5.72	6.00
	***TR-07***	37.56	28.79	21.10	17.76	12.18	9.54	9.12	9.90	9.56	11.25	9.11	11.11
	***TA-28***	43.38	29.17	19.36	18.13	8.15	4.74	5.22	4.57	4.32	5.62	5.33	5.33
	***Zendura A***	47.60	49.88	44.23	49.24	40.48	43.73	46.31	47.47	44.36	50.18	55.59	48.74
	***Zendura FLX***	23.53	21.55	19.28	20.63	19.90	21.50	23.40	19.53	18.99	20.30	26.63	25.57
***3***	***TC-85***	39.85	27.50	21.88	16.80	9.86	6.84	6.50	5.77	7.05	8.16	6.97	7.30
	***TR-07***	48.78	35.91	26.58	22.16	14.83	11.92	11.16	12.35	11.79	13.26	11.27	13.48
	***TA-28***	56.34	36.51	25.02	22.16	9.86	5.81	6.21	5.61	5.31	6.68	6.36	6.42
	***Zendura A***	59.87	65.70	58.26	64.91	53.75	58.47	62.13	63.53	59.63	66.17	73.86	65.23
	***Zendura FLX***	29.61	27.00	25.55	25.91	25.97	27.38	30.06	25.57	25.36	26.23	33.07	32.91

### Young’s modulus

**Fig. 4:**
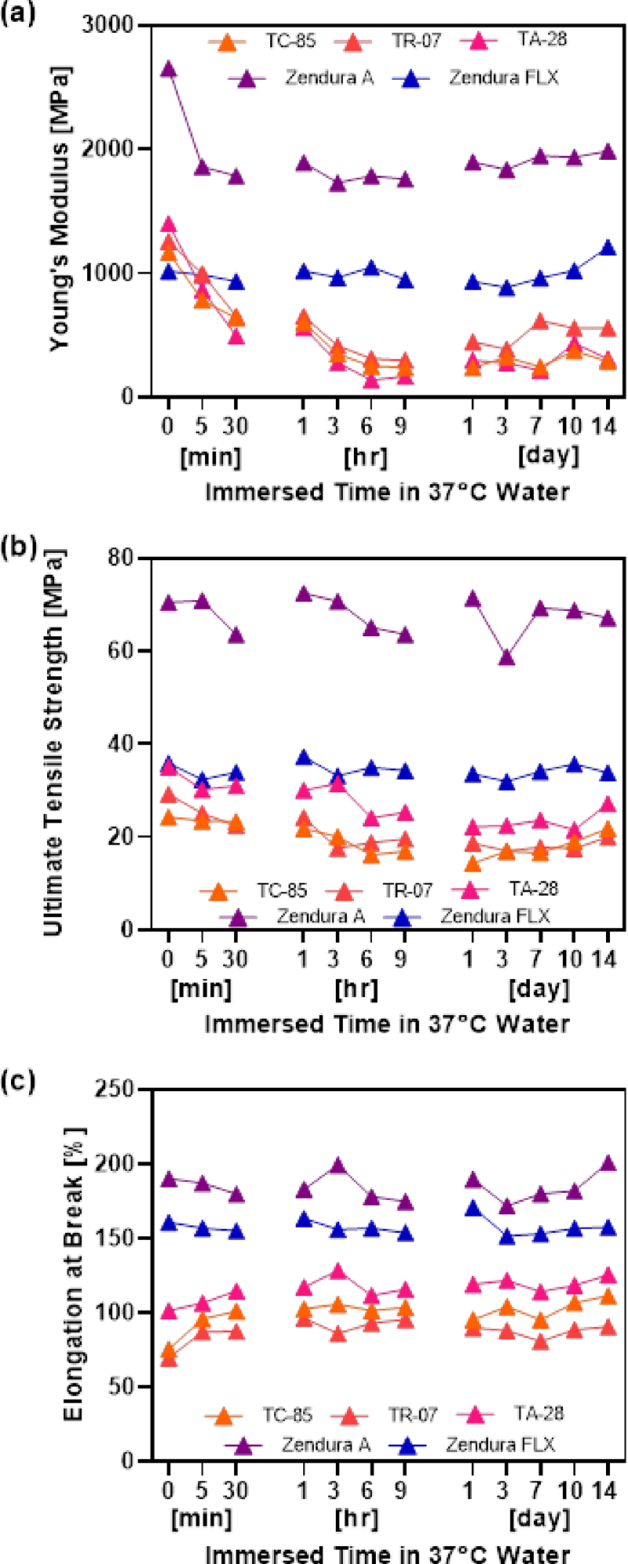
Tensile test results of five different clear aligner materials (TC-85, TR-07, TA-28, Zendura A, Zendura FLX) at various immersion times in 37 °C distilled water. **(a)** Young’s modulus (MPa), **(b)** ultimate tensile strength (MPa), **(c)** elongation at break (%).

The Young’s modulus (MPa) was measured to assess the stiffness of the aligner materials over time at 37 °C, as shown in Fig. 4(a) and Table 3. Zendura A and Zendura FLX exhibited minimal changes in stiffness throughout the immersion period, with that of Zendura A starting at 2654.8 MPa and slightly decreasing to 1986.8 MPa after 14 d. The stiffness of Zendura FLX remained relatively stable, ranging from 1016.9 to 1212.3 MPa.

In contrast, TC-85, TR-07, and TA-28 exhibited significant reductions in stiffness over time owing to their thermally responsive behavior. Over the 14-d period, TC-85 exhibited a reduction from 1169.8 to 289.58 MPa, TR-07 exhibited a reduction from 1259.4 to 560.18 MPa, and TA-28 exhibited a reduction from 1401.6 to 311.1 MPa.

Intra-material analysis (Friedman test) revealed no significant changes over time in Zendura A and Zendura FLX, whereas direct-printed resins exhibited significant differences at early time points.

### Ultimate tensile strength

In this study, thermoformed materials exhibited higher UTS values than direct-printed resins.

For all immersion times, Zendura A showed the highest UTS (58.76–72.44 MPa), indicating high stability at body temperature. Then, it was followed by Zendura FLX (31.97–37.29 MPa), corresponding to roughly half the strength of Zendura A and remaining within a similar magnitude range to the direct-printed materials. Among the direct-printed materials, TA-28 exhibited intermediate UTS values (21.52–35.06 MPa), whereas TR-07 (17.00–29.20.00.20 MPa), then TC-85 (14.38–24.29 MPa) showed the lowest UTS.

The mean UTS values are shown in Fig. 4(b) and Table 4.

### Elongation at break

The elongation at break (%) shown in Fig. 4(c) and Table 5, was measured to assess the stretchability of the aligner materials. Zendura A exhibited the highest elongation values, starting at 190.2% and increasing slightly to 201.12% at 14 d, demonstrating minimal fluctuation over time. Zendura FLX exhibited similar stability, maintaining elongation values between 151.7% and 170.8% over the 14-d immersion period.

TC-85, TR-07, and TA-28 exhibited significant increases in elongation, particularly during the early immersion stages. The elongation of TC-85 increased from 75.54% at time 0 to 111.5% at day 14; that of TR-07 increased from 69.78% to 90.73%; and that of TA-28 steadily increased from 101.6% to 125.6% by day 14.

Significant differences were consistently observed among Zendura A, TC-85, and TR-07 (*p* < 0.0001). Intra-material analysis indicated that Zendura A and Zendura FLX maintained consistent elongation values, whereas TC-85 and TR-07 exhibited greater variation with significant differences, particularly during the initial immersion period.

**Table 3:** Young’s modulus (MPa) of clear aligner materials with varied water immersion times.

Clear Aligner Materials	Immersed Time in 37° C Water Bath											
	0 min	5 min	30 min	1 h	3 h	6 h	9 h	1 day	3 days	7 days	10 days	14 days
***TC-85***	1169.8 ± 118.53	784.11 ± 43.626	637.56 ± 68.735	603.77 ± 79.507	349.46 ± 62.975	252.04 ± 29.291	240.16 ± 36.644	239.25 ± 44.943	325.1 ± 40.705	245.87 ± 62.524	374.83 ± 53.255	289.58 ± 18.512
***TR-07***	1259.4 ± 156.82	995.84 ± 152.62	655.49 ± 73.678	653.49 ± 125.27	415.44 ± 42.687	312.61 ± 35.909	298.89 ± 55.99	448.73 ± 82.66	387.61 ± 131.03	619.4 ± 188.66	557.3 ± 38.996	560.18 ± 84.80
***TA-28***	1401.6 ± 126.22	867.08 ± 118.02	496.37 ± 149.6	564.28 ± 121.32	280.32 ± 61.538	137.99 ± 10.506	170.25 ± 54.908	299.42 ± 55.547	277.48 ± 70.585	217.15 ± 86.912	437.46 ± 50.815	311.1 ± 128.15
***Zendura A***	2654.8 ± 1669.8	1859.6 ± 137.07	1786.5 ± 142.44	1894 ± 108.11	1730.3 ± 243.54	1786.8 ± 200.27	1761.2 ± 192.39	1898.2 ± 93.855	1836.4 ± 128.64	1949.8 ± 175.14	1936.5 ± 85.741	1986.8 ± 303.98
***Zendura FLX***	1016.9 ± 118.53	988.57± 43.626	937.69 ± 68.735	1018.9 ± 79.507	966.74 ± 62.975	1048.3 ± 29.291	949.77 ± 36.644	934.55 ± 44.943	885.96 ± 40.705	962.84 ± 62.524	1023.4 ± 53.255	1212.3 ± 18.512

**Table 4:** Ultimate tensile strength (MPa) of different clear aligner materials with varied water immersion times.

Clear Aligner Materials	Immersed Time in 37° C Water Bath											
	0 min	5 min	30 min	1 h	3 h	6 h	9 h	1 day	3 days	7 days	10 days	14 days
***TC-85***	24.29 ± 2.681	23.44 ± 2.909	23.34 ± 3.219	21.81 ± 3.298	20.07 ± 2.439	16.19 ± 2.497	16.96 ± 2.017	14.38 ± 1.794	16.96 ± 3.003	16.64 ± 1.723	19.00 ± 4.468	21.84 ± 3.306
***TR-07***	29.2 ± 0.9126	25.12 ± 3.855	22.45 ± 1.848	24.18 ± 1.139	17.6 ± 1.747	18.83 ± 2.870	19.61 ± 3.756	18.70 ± 2.288	17.00 ± 1.769	17.79 ± 3.243	17.54 ± 2.101	20.04 ± 3.251
***TA-28***	35.06 ± 3.080	30.25 ± 1.973	31.00 ± 5.384	30.06 ± 3.307	31.54 ± 7.144	24.15 ± 2.850	25.3 ± 1.499	22.23 ± 4.316	22.47 ± 3.890	23.62 ± 2.421	21.52 ± 2.442	27.2 ± 5.159
***Zendura A***	70.46 ± 5.577	70.9 ± 7.159	63.58 ± 11.26	72.44 ± 10.95	70.73 ± 11.00	65.15 ± 4.189	63.59 ± 7.269	71.37 ± 5.890	58.76 ± 6.075	69.31 ± 10.20	68.74 ± 4.880	67.176 ± 7.500
***Zendura FLX***	35.9 ± 2.681	32.39 ± 2.909	33.97 ± 3.219	37.29 ± 3.298	33.19 ± 2.439	34.95 ± 2.497	34.3 ± 2.017	33.54 ± 1.794	31.97 ± 3.003	34.14 ± 1.723	35.69 ± 4.468	33.87 ± 3.306

**Table 5:** Elongation at break (%) of clear aligner materials with varied water immersion times.

Clear Aligner Materials	Immersed Time in 37° C Water Bath											
	0 min	5 min	30 min	1 h	3 h	6 h	9 h	1 day	3 days	7 days	10 days	14 days
***TC-85***	75.54 ± 18.5	95.81 ± 8.199	101.1 ± 7.918	102.7 ± 8.562	105.7 ± 5.724	101.5 ± 5.884	103.7 ± 5.438	95.42 ± 4.996	104.1 ± 10.40	95.39 ± 5.272	106.8 ± 10.25	111.5 ± 4.312
***TR-07***	69.78 ± 8.435	87.18 ± 11.08	87.58 ± 6.958	96.36 ± 1.863	86.21 ± 6.704	93.10 ± 8.623	95.48 ± 9.68	89.92 ± 8.187	88.2 ± 4.671	80.97 ± 8.816	88.62 ± 6.753	90.73 ± 8.686
***TA-28***	101.6 ± 15.97	106.6 ± 4.876	114.7 ± 10.75	117.3 ± 7.008	128.6 ± 10.55	111.6 ± 4.768	115.8 ± 1.674	119.1 ± 8.096	121.9 ± 9.031	114.3 ± 4.513	118.4 ± 6.801	125.6 ± 6.849
***Zendura A***	190.2 ± 11.97	187.2 ± 13.53	179.8 ± 26.38	183 ± 19.98	199.4 ± 22.95	178.1 ± 13.78	174.9 ± 22.98	189.7 ± 17.07	171.9 ± 12.48	180 ± 25.30	181.9 ± 13.42	201.12 ± 2.370
***Zendura FLX***	160.8 ± 18.50	156.9 ± 8.199	155.1 ± 7.918	163.5 ± 8.562	156 ± 5.724	157 ± 5.884	153.9 ± 5.438	170.8 ± 4.996	151.7 ± 10.40	153.3 ± 5.272	156.8 ± 10.25	157.7 ± 4.312

## Discussion

The primary objective of this study was to compare the mechanical behavior of thermoformed and direct-printed aligner materials under prolonged 37 °C water immersion up to 14 days, reflecting controlled thermo-hydric exposure over a typical aligner wear cycle. Within this timeframe, the direct-printed resins TC-85, TR-07, and TA-28 were evaluated with the conventional thermoformed materials, Zendura A (monolayer TPU) and Zendura FLX (multilayer TPU).

### Post-manufacturing thickness considerations

Aligners undergo thickness changes during fabrication, and thus the clinically relevant thickness is the post-manufacturing dimension.

In this study, thermoforming of 0.76 mm sheets resulted in substantial thinning due to heat-induced stretching, yielding mean final thicknesses of 0.414 mm for Zendura A (45.5% reduction) and 0.381 mm for Zendura FLX (50.5% reduction).

Conversely, directly printed materials showed an increase from their design value, yielding final thicknesses of 0.560–0.585 mm, representing a 40–46% increase from their 0.40-mm design thickness owing to the layer-by-layer additive printing process.

These fabrication-dependent thickness trends are consistent with previous reports and reflect inherent differences between thermoforming-induced stretching and additive manufacturing process^8,34^. Accordingly, the final dimensional measurements should be considered when interpreting material behavior into clinical performance.

### Tensile force at 1% − 3% strain

With no hydro-thermal exposure, Zendura A exhibited the highest tensile force for all recorded strain levels, followed by TA-28, TR-07, TC-85, and Zendura FLX, respectively, with Zendura FLX showing the lowest tensile force in the unexposed state.

With hydro-thermal exposure, TC-85, TR-07, and TA-28 demonstrated significant temperature sensitivity. The direct-printed resins exhibited a reduction in force with water at body temperature, with the most pronounced decrease within the first hour and stabilization thereafter. After 1 h, all three direct printed materials show lower tensile forces than thermoformed materials. This behavior is attributed to the thermally responsive viscoelastic nature of urethane acrylate-based shape memory polymers (SMPs)^2,15^. The observed ~ 1 h stabilization period is consistent with the reported > 95% shape recovery time of TC-85 at body temperature^2^. After 2 weeks of immersion, TA-28 showed the greatest force reduction, reflecting its higher temperature sensitivity and elastic response, whereas TR-07 showed the least change, consistent with its retainer-oriented design with weaker shape memory activation. Overall, the direct printed resins maintained reduced force levels at body temperature, supporting a more comfortable fit within biomechanically desired orthodontic force ranges^33,35^.

In contrast, Zendura A and Zendura FLX exhibited more consistent tensile forces, with < 15 N variation over 2-week period at 3% strain. Zendura A maintained tensile force levels within 53–74 N and Zendura FLX remained within 25–34 N, approximately the force of Zendura A, consistent with its multilayer design incorporating an elastomeric core for increased flexibility. Unlike direct-printed resins, thermoformed materials showed minimal early force attenuation and exhibited delayed force fluctuations after 7 days. This stability is associated with their thermoplastic composition and more rigid force-delivery profiles.

Previous studies support our findings. Atta et al. reported that Zendura A exhibits the highest force, followed by Zendura FLX and TC-85, with an initial force reduction during the first hour^15^. Lee et al. demonstrated that TC-85 undergoes rapid initial stress relaxation due to its amorphous-phase crystallization, whereas PET-G maintains nearly constant stress over 13 repeated cycles^2^. TC-85 retained a residual force of 1.0 N compared with 11.39 N for PET-G^2^. Reported orthodontic force requirements range from 0.068 to 1.2 N^2,9,33,35^. Prior studies further indicate that direct-printed aligner (TC-85) designed with 0.1–0.3 mm tooth movement generates stabilized forces 0.76–1.57 N, whereas thermoformed aligner (ATMOS) showed 4.73–15.04 N^13^, with some reports exceeding 20 times the physiologic range^36^.

### Young’s modulus and stiffness response

The Young’s modulus is a measure of the intrinsic stiffness of a material. It reflects the balance between force delivery and flexibility, required for effective yet comfortable orthodontic movement.

The trends in Young’s modulus closely paralleled those observed for tensile force.

Shape memory materials, TC-85, TR-07, and TA-28, exhibited a reduction in Young’s modulus after water immersion. On the other hand, thermoformed materials, Zendura A and Zendura FLX, exhibited minimal stiffness changes under identical conditions, owning to their thermoplastic behavior, which do not exhibit significant changes in tensile properties after intraoral exposure^37^.

Previous studies on TC-85^2,15^ also support that TC-85 has lower rigidity than other clear aligner materials such as PETG and TPU. In the present study, the 3D-printed resins initially demonstrated Young’s moduli similar to that of Zendura FLX (multilayer TPU) in the range of 1100–1400 MPa, but following immersion in 37 °C water, TC-85 and TA-28 rapidly stabilized at lower modulus values (200–600 MPa within 3 h), owing to their thermal responsive polymer network.

The temperature-dependent softening of shape memory materials is consistent with findings by Choi et al.^38^, highlighting its unique stress relaxation capability and shape memory features. The increased flexibility allows direct-printed resins to deliver reduced forces at body temperature, aligning with biomechanically recommended orthodontic force ranges (0.2–2.5 N)^2,31–33^.

### Ultimate tensile strength and durability

Ultimate tensile strength (UTS) reflects the material’s resistance to fracture under excessive or accidental loading rather than its orthodontic force delivery, as aligners typically undergo only small elastic deformation (< 3%) during clinical use^9^.

The higher UTS observed in thermoformed materials, particularly Zendura A, indicates superior resistance to fracture during insertion and removal compared with direct-printed resins. Zendura FLX exhibited intermediate UTS values, approximately half those of Zendura A, consistent with its multilayer design intended to balance flexibility and strength.

Among the direct-printed materials, TA-28 demonstrated UTS values comparable to Zendura FLX, whereas TR-07 and TC-85 showed lower UTS values, consistent with their higher flexibility and shape-memory-driven compliance.

Overall, all materials maintained sufficient structural integrity under prolonged thermo-hydric exposure, supporting their suitability for clinical conditions.

### Elongation at break and flexibility

Direct-printed materials (TC-85, TR-07, and TA-28) showed increased elongation at break with prolonged water immersion, with TC-85 exhibiting the most significant increase. This behavior reflects the temperature-responsive viscoelastic nature of these shape memory resins and indicates enhanced flexibility under thermo-hydric exposure, which may contribute to improved comfort during treatment.

Consistent with our findings, Choi, J. Y^38^. reported that TC-85 experiences gradual increase in elongation with rising temperature. However, it should be noted that elastic deformations exceeding 3% are not typically encountered during clinical aligner use, as aligners are generally designed to achieve 0.1–0.3 mm of tooth movement, corresponding to 1–3% strain.

Although the measured elongation does not directly represent clinical deformation levelts, all the materials exceeded the minimum elongation thresholds reported for polymeric dental applications^39^, supporting their mechanical robustness and clinical functionality.

### Limitations

This study has several limitations that should be considered when interpreting the results. First, the present setup evaluated uniaxial tensile behavior of flat specimens, which may not fully replicate the complex intraoral loading and geometry of clinical aligners. Second, inherent difference in fabrication methods and material behavior and lack of standard testing methods make it difficult to compare two differently fabricated devices directly. Third, as an *in vitro* study, direct extrapolation to clinical settings is limited, as real-world variables such as fluctuating temperature, humidity, saliva, and complex biomechanical forces may affect material performance.

Future research should include more complex oral conditions to examine more factors for *in vitro* aging and explore the mechanical effect of lower deformation ranges on materials, along with the impact of material thickness and design on mechanical properties. Additionally, clinical trials evaluating the performance of new direct-printed resins in patients are recommended to provide a more comprehensive understanding of their effectiveness and durability in orthodontic treatment.

Clinically relevant aligner deformation is likely below 1%. Future studies should focus on lower strain levels (e.g., 0.25% or 0.5%) to further refine orthodontic force characterization.

### Clinical implications

Thermoformed TPU materials and direct-printed resins operate on different force-delivery principles: thermoformed materials tend to retain higher stiffness and force magnitudes, whereas direct-printed resins exhibit temperature-responsive viscoelastic behavior that reduces force and stiffness at body temperature.

From a clinical perspective, higher stiffness may support more consistent force expression but may compromise comfort during insertion and removal and potentially affect patient compliance during repeated insertion–removal cycles^7,9^.

Conversely, the increased flexibility of direct-printed resins at 37 °C may improve comfort, while the observed stabilization of mechanical responses up to 14 days suggests their force delivery may remain sustained over a typical aligner wear period^23^.

Furthermore, direct printing enables digital thickness customization for force modulation; however, additional studies are needed to quantify how thickness and design variations influence force delivery and durability across clinically relevant geometries^40,41^.

## Conclusions

The main conclusions of the study are as follows:

- At 1%–3% strain, direct-printed aligner materials (TC-85, TR-07, and TA-28) exhibited significant temperature sensitivity, with reduced forces at 37 °C, whereas thermoformed materials (Zendura A and Zendura FLX) exhibited more consistent and higher tensile force levels.
- Young’s modulus followed a similar trend. Direct-printed aligner materials softened and stabilized at lower stiffness after immersion, while thermoformed materials, particularly Zendura A, remained comparatively stiff.
- Thermoformed mateirals exhibited higher UTS than direct-printed resins, with Zendura A showing the highest values.
- Elongation at break increased for direct-printed materials with 37 °C water immersion, whereas thermoformed mateirals remained relatively stable.
- Overall, prolonged 37 °C water immersion highlighted two distinct mechanical profiles: thermoformed TPU materials retained higher force and stiffness, whereas direct-printed resins became more flexible with reduced force magnitudes.

These findings suggest that the lower force magnitudes and increased flexibility observed in direct-printed aligner materials may not reflect mechanical inferiority but rather imply a potentially strategic design feature in clinical orthodontics. Owing to their temperature-responsive and shape memory characteristics, these materials are expected to deliver gentle and sustained orthodontic forces within a physiologically desirable range during wear. Moreover, the ability to digitally customize the thickness and aligner geometry may allow more predictable and efficient treatment strategies in the future. However, clinical studies are required to validate their long-term performance under functional intraoral conditions.

## Data Availability

The datasets used and/or analysed during the current study available from the corresponding author on reasonable request.
