# Correction: Mechanical properties of thermoformed and direct-printed aligner materials after immersion in 37 °C water: a 14-day *in vitro* study

**DOI:** 10.1038/s41598-026-62169-z

**Published:** 2026-07-15

**Authors:** Rodrigo Oyonarte, Isabel Margarita Lagos, Francisca Vidaurre L., Tomás Parada B., Alberto del Real, Soonho Jang, Harim Jeong, Jiho Lee, Jinhong Min, Tarek M. Elshazly, Jung-Yul Cha, Hoon Kim

**Affiliations:** 1 Facultad de Odotología, Universidad de los Andes, Monseñor Alvaro del Portillo, Las Condes, Santiago, 12455 Chile; 2 Graphy Inc., Graphy R&D Center, Seoul, 08501 Republic of Korea; 3 Oral Technology Department, Dental School, University Hospital Bonn, Welschonnenstr. 17, 53111 Bonn, Germany; 4 Department of Orthodontics, Institute of Craniofacial Deformity, College of Dentistry, Yonsei University, 50 1 Yonsei ro, Seodaemun gu, Seoul, 03722 Korea; 5 Research Institute of Agriculture and Life Sciences, College of Agriculture & Life Sciences, Seoul National University, Seoul, 08826 Republic of Korea

Correction to: *Scientific Reports* 10.1038/s41598-026-36723-8, published online 21 January 2026

The original version of this Article contains errors in Table 2, where the strain values were incorrectly labelled. Specifically, the values were erroneously reported as 0.1, 0.2, and 0.3 now read 1, 2, and 3, which correspond to the intended 1–3% strain levels used in the mechanical testing protocol. The corrected and uncorrected table appear below.

Incorrect Table 2:

**Table:** 

Strain (%)	Clear Aligner Materials	Immersed Time in 37° C Water Bath											
		0 min	5 min	30 min	1 h	3 h	6 h	9 h	1 day	3 days	7 days	10 days	14 days
***0.1***	***TC-85***	18.62	13.58	10.30	8.54	5.34	3.85	3.65	2.88	3.87	4.97	3.78	4.04
	***TR-07***	20.96	17.27	12.23	10.68	7.91	6.06	6.09	6.10	6.10	7.85	5.89	7.24
	***TA-28***	23.84	17.37	10.91	11.16	5.50	3.38	3.88	3.11	2.91	4.14	3.70	3.67
	***Zendura A***	28.09	27.01	23.46	26.46	21.37	22.89	24.12	25.09	23.62	28.41	31.10	26.26
	***Zendura FLX***	13.08	11.97	9.62	11.53	10.62	11.79	12.70	10.42	9.95	11.59	16.28	14.36
***0.2***	***TC-85***	31.58	22.16	17.39	13.66	8.10	5.63	5.34	4.54	5.83	6.91	5.72	6.00
	***TR-07***	37.56	28.79	21.10	17.76	12.18	9.54	9.12	9.90	9.56	11.25	9.11	11.11
	***TA-28***	43.38	29.17	19.36	18.13	8.15	4.74	5.22	4.57	4.32	5.62	5.33	5.33
	***Zendura A***	47.60	49.88	44.23	49.24	40.48	43.73	46.31	47.47	44.36	50.18	55.59	48.74
	***Zendura FLX***	23.53	21.55	19.28	20.63	19.90	21.50	23.40	19.53	18.99	20.30	26.63	25.57
***0.3***	***TC-85***	39.85	27.50	21.88	16.80	9.86	6.84	6.50	5.77	7.05	8.16	6.97	7.30
	***TR-07***	48.78	35.91	26.58	22.16	14.83	11.92	11.16	12.35	11.79	13.26	11.27	13.48
	***TA-28***	56.34	36.51	25.02	22.16	9.86	5.81	6.21	5.61	5.31	6.68	6.36	6.42
	***Zendura A***	59.87	65.70	58.26	64.91	53.75	58.47	62.13	63.53	59.63	66.17	73.86	65.23
	***Zendura FLX***	29.61	27.00	25.55	25.91	25.97	27.38	30.06	25.57	25.36	26.23	33.07	32.91

Corrected Table 2.

**Table:** 

Strain (%)	Clear Aligner Materials	Immersed Time in 37° C Water Bath											
		0 min	5 min	30 min	1 h	3 h	6 h	9 h	1 day	3 days	7 days	10 days	14 days
***1***	***TC-85***	18.62	13.58	10.30	8.54	5.34	3.85	3.65	2.88	3.87	4.97	3.78	4.04
	***TR-07***	20.96	17.27	12.23	10.68	7.91	6.06	6.09	6.10	6.10	7.85	5.89	7.24
	***TA-28***	23.84	17.37	10.91	11.16	5.50	3.38	3.88	3.11	2.91	4.14	3.70	3.67
	***Zendura A***	28.09	27.01	23.46	26.46	21.37	22.89	24.12	25.09	23.62	28.41	31.10	26.26
	***Zendura FLX***	13.08	11.97	9.62	11.53	10.62	11.79	12.70	10.42	9.95	11.59	16.28	14.36
***2***	***TC-85***	31.58	22.16	17.39	13.66	8.10	5.63	5.34	4.54	5.83	6.91	5.72	6.00
	***TR-07***	37.56	28.79	21.10	17.76	12.18	9.54	9.12	9.90	9.56	11.25	9.11	11.11
	***TA-28***	43.38	29.17	19.36	18.13	8.15	4.74	5.22	4.57	4.32	5.62	5.33	5.33
	***Zendura A***	47.60	49.88	44.23	49.24	40.48	43.73	46.31	47.47	44.36	50.18	55.59	48.74
	***Zendura FLX***	23.53	21.55	19.28	20.63	19.90	21.50	23.40	19.53	18.99	20.30	26.63	25.57
***3***	***TC-85***	39.85	27.50	21.88	16.80	9.86	6.84	6.50	5.77	7.05	8.16	6.97	7.30
	***TR-07***	48.78	35.91	26.58	22.16	14.83	11.92	11.16	12.35	11.79	13.26	11.27	13.48
	***TA-28***	56.34	36.51	25.02	22.16	9.86	5.81	6.21	5.61	5.31	6.68	6.36	6.42
	***Zendura A***	59.87	65.70	58.26	64.91	53.75	58.47	62.13	63.53	59.63	66.17	73.86	65.23
	***Zendura FLX***	29.61	27.00	25.55	25.91	25.97	27.38	30.06	25.57	25.36	26.23	33.07	32.91

The article has been corrected.

